# Near-Infrared Irradiation Non-thermally Affects Subcutaneous Adipocytes and Bones

**Published:** 2011-03-09

**Authors:** Yohei Tanaka, Kiyoshi Matsuo, Shunsuke Yuzuriha

**Affiliations:** Department of Plastic and Reconstructive Surgery, Shinshu University School of Medicine, Matsumoto, Nagano 390-8621, Japan

## Abstract

**Objective:** We previously reported that near-infrared irradiation simulating solar near-infrared with pre- and parallel-irradiational cooling can penetrate the skin and non-thermally affects dermis, superficial muscles, and so forth. To clarify the possible effect of NIR irradiation on other subcutaneous tissues, we evaluated how near-infrared non-thermally affects subcutaneous adipocytes and bones in rats. **Methods:** The central back tissues of rats were irradiated with a specialized near-infrared device that simulates solar radiation. The total energy emitted was equivalent to approximately 8.75 hours of sunbathing in North America. Histological evaluation was performed on subcutaneous adipocytes and the spinous process of the near-infrared-irradiated rat and compared with non-irradiated controls. **Results:** Subcutaneous and bone marrow adipocytes, CD34-positive hematopoietic stem cells in bone marrow, and the cortical bone mass were all significantly increased, whereas the bone marrow cell number was significantly decreased following near-infrared irradiation. Apoptotic cells were detected in the bone marrow at postirradiation days 7 and 30 but were not detected at day 60 or in the controls. Bone marrow cell numbers recovered gradually, whereas the increase in subcutaneous and bone marrow adipocytes, CD34-positive hematopoietic stem cells in bone marrow, and cortical bone mass remained elevated even at day 180. **Conclusions:** Near-infrared irradiation that simulated solar radiation non-thermally affected subcutaneous adipocytes and bones in rats. It induced putative, non-thermal damage of bone marrow, which was mediated by apoptosis. However, it increased subcutaneous and bone marrow adipocytes, CD34-positive hematopoietic stem cells in bone marrow, and cortical bone mass.

Near-infrared (NIR) is an electromagnetic radiation that simultaneously exhibits both wave and particle properties and is strongly absorbed by water, hemoglobin, or myoglobin. We previously reported that NIR irradiation simulating solar NIR at specific wavelengths with pre- and parallel-irradiational cooling can penetrate the skin and non-thermally affect dermis,^1^^-^3 superficial muscles,^4^,^5^ and so forth.^6^ To clarify the possible effect of NIR irradiation on other subcutaneous tissues, we sought to evaluate how NIR non-thermally affects subcutaneous adipocytes and bones in rats.

## MATERIALS AND METHODS

### Animals

Thirty-five male Wistar rats (*Rattus norvegicus albinu*s) weighing 360 to 440 g were used. Experiments were performed in a temperature-controlled environment (24°C ± 1.5°C) under a 12-hour light-dark cycle with free access to water and standard rat chow. All animals were treated humanely and in compliance with the recommendations of the animal care local committee. The study was approved by our institutional ethics committee for animal experiments. Animals were anesthetized with an intra-abdominal dose of sodium pentobarbitone (50 mg/kg, IP) and were sacrificed by intracardiac administration of ketamine (150 m/kg) upon completion of the experiment.

### NIR irradiation

Near-infrared irradiation was generated using a broadband NIR source (Titan; Cutera, Brisbane, Calif). The device emitted a NIR spectrum ranging from 1100 to 1800 nm, filtering wavelengths between 1400 and 1500 nm, thereby simulating solar radiation. This allows us to deliver NIR without the wavelengths that are strongly absorbed by water and hemoglobin and allow safe delivery of NIR energy deep into the tissue. The horizontal spot size of irradiation was 10 × 30 mm. The system delivered energy with a fluence range from 5 to 56 J/cm^2^ using continuous energy single irradiation pulses ranging from 4 to 10 seconds. The sapphire contact cooling tip was set to a fixed temperature of 20°C to provide contact cooling. Pre- and parallel-irradiational cooling of the superficial layers was accomplished using a temperature-controlled sapphire window, which further prevented excessive superficial heating.

Thirty-five rats were either irradiated (*n* = 25) or not (control; *n* = 10). The center of the dorsal portion of the irradiated rats was subjected to 3 rounds of irradiation at 40 J/cm^2^ on days 0, 7, and 14 without application of topical anesthesia. One round of irradiation consisted of 2 passes of NIR irradiation.

### Histological evaluation

Specimens, which included the spinous process of the sixth lumbar vertebra and the overlying subcutaneous tissues, were isolated from the experimental group (5 rats per time point) at 7, 30, 60, 90, and 180 days after the final dose of irradiation (days 7, 30, 60, 90, and 180, respectively). Control samples were only isolated at days 0 and 180 (5 rats per time point). The specimens were fixed in 20% neutral buffered formalin, processed for paraffin embedding, and serially sectioned along the sagittal plane (3- to 4 µm thickness)

Tissue sections were stained with hematoxylin and eosin as well as with an anti-CD34 antibody for immunohistochemical analysis to determine CD34-positive cells in subcutaneous tissue and hematopoietic stem cells in bone marrow. CD34 staining positive cells in subcutaneous tissue are not an indicator for CD34 positive adipose-derived stem cells. Thus, we did not calculate the number of CD34-positive cells on the panniculus carnosus in this study. The transferase-mediated dUTP nick-end labeling (TUNEL) assay was used to stain apoptotic cells.

The percentage of the area occupied by subcutaneous adipocytes was calculated for all time points in an area 0.5 mm high × 2 mm wide on the panniculus carnosus, which was located in the middle of spinous process. The percentage of the area occupied by bone marrow adipocytes, the cortical bone mass, as well as hematopoietic bone marrow cell counts and CD34-positive stem cell counts was calculated in a superficial area 1.5 mm deep in the spinous process.

Images were scanned and quantified in 5 representative fields per section and subsequently averaged to obtain a final score. The sections were photographed under a BIOREVO BZ-9000 microscope (Keyence, Osaka, Japan). The digital photographs were processed using Adobe Photoshop (Adobe, San Jose, Calif).

### Statistical analyses

The differences between groups at each time point were examined for statistical significance using the Mann-Whitney U test. *P* <.05 was set as a cutoff for statistical significance. Data are represented as means±standard deviation.

## RESULTS

**Figure 1:**
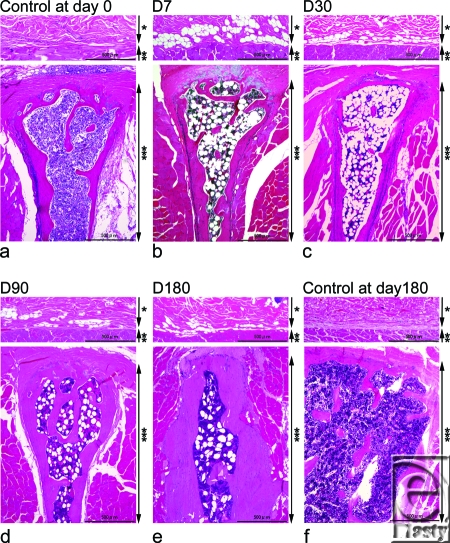
Hematoxylin and eosin staining (a-f). The dermis, subcutaneous tissues, and panniculus carnosus are shown above, and the spinous process is shown below for each experimental point. The control at day 0 (a), and experimental samples at day 7 (b), day 30 (c), day 90 (d), day 180 (e), and the control at day 180 (f) are indicated. Scale bars = 500 µm. An asterisk indicates a layer of subcutaneous tissue where adipocytes have been induced, 2 asterisks indicate the panniculus carnosus, and 3 asterisks indicate the evaluated spinous process.

**Figure 2:**
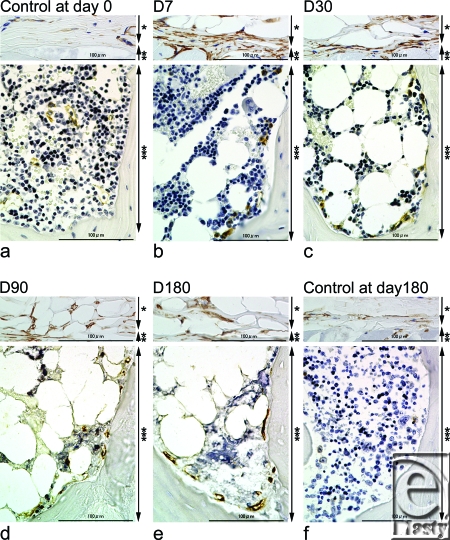
Immunohistochemical staining with an anti-CD34 antibody (a-f). The dermis, subcutaneous tissues, and panniculus carnosus are shown above, and the spinous process is shown below for each experimental time point. CD34-positive cells (stained brown) in the control at day 0 (a), experimental samples at day 7 (b), day 30 (c), day 90 (d), day 180 (e), and the control at day 180 (f) are indicated. Scale bars = 100 µm. An asterisk indicates a layer of subcutaneous adipocytes and subcutaneous tissue, 2 asterisks indicate the degenerated panniculus carnosus and 3 asterisks indicate an enlarged part of the evaluated spinous process.

**Figure 3:**
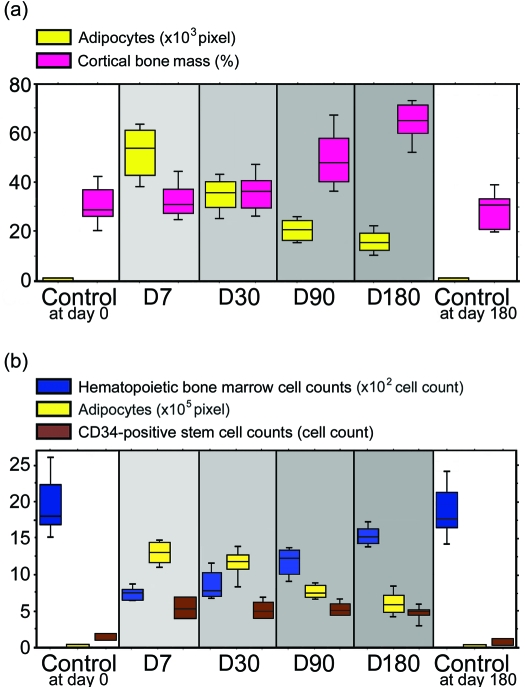
(a) Chronological changes in the percentage of the area occupied by subcutaneous adipocytes in an area 0.5 mm high × 2 mm wide on the panniculus carnosus, which was located in the middle of the spinous process, are indicated in yellow. Chronological changes in the percentage of the area occupied by the cortical bone mass in an area of superficial depth (1.5 mm) in the spinous process are indicated in red. (b) Chronological changes in the hematopoietic bone marrow cell counts are indicated in blue, the percentage of the area occupied by bone marrow adipocytes in yellow, and the CD34-positive stem cell counts in brown. All were located in an area of superficial depth (1.5 mm) in the spinous process.

Subcutaneous adipocytes on the panniculus carnosus overlying the spinous process were abruptly induced at day 7 and subsequently showed a gradual decrease (Figs 1–3). Statistically significant increases were observed at days 7, 30, 90, and 180, compared with non-irradiated controls at days 0 and 180 (*P* <0.05). Subcutaneous adipocytes on the panniculus carnosus overlying the spinous process were not observed in the non-irradiated controls at either time point.

Bone marrow adipocytes were also induced dramatically at day 7 and after which numbers gradually decreased (Figs 1–3). Significant increases were observed at days 7, 30, 90, and 180, compared with the non-irradiated controls at days 0 and 180 (*P* <0.05).

Cortical bone mass increased steadily over the 6-month period (Figs 1 and 3). Significant increases in cortical bone mass were only observed at days 90 and 180 compared with non-irradiated controls (*P* <0.05). Statistically significant differences were not achieved between control samples at day 0 and experimental samples at either days 7 or 30 (*P* = 0.8340 and *P* = 0.3472, respectively), as well as control samples at days 0 and 180 (*P* = 0.7540).

Hematopoietic bone marrow cell counts in the irradiated groups decreased significantly at day 7 and then increased gradually from day 30 to day 180 (Figs 1–3). Significant decreases were observed between samples at days 7, 30, 90, and 180 and the non-irradiated controls at days 0 and 180 (*P* <0.05) (Figs 1–3).

CD34-positive cells on the panniculus carnosus appeared to increase at day 7 and then slowly decrease (Fig 2). Numbers of CD34-positive stem cells in the bone marrow were dramatically increased at day 7 and this increase persisted till day 180. Significant increases in cell number were observed at days 7, 30, 90, and 180 compared with the non-irradiated controls at days 0 and 180 (*P* <0.05) (Figs 2 and 3—lower panel). A majority of CD34-positive stem cells in bone marrow were observed at the inner surface of the bone cortex (Fig 2).

**Figure 4:**
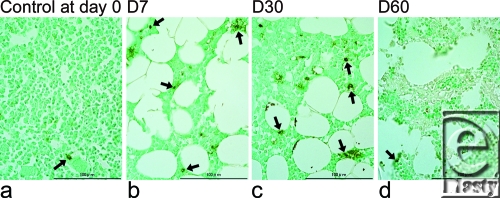
Transferase-mediated dUTP nick-end labeling (TUNEL) staining of apoptotic cells (a-d). TUNEL staining of bone marrow was almost completely negative in controls at day 0 (a) and experimental samples at day 60 (d), but positive at day 7 (b) and day 30 (c). Representative TUNEL positive cells are indicated by black arrows.

TUNEL staining of the bone marrow showed almost no apoptotic cells in control samples at day 0 or irradiated samples at day 60. However, positive staining was seen at days 7 and 30 (Fig 4). There were no evidences of necrosis, including inflammation and hyperplasia of fibroblasts or lymphocytes, in irradiated samples obtained at days 7 and 30.

## DISCUSSION

Because of its dual wave and particulate properties, NIR irradiation is able to penetrate the thin layer of skin (1000-1300 µm in this study) and minimal subcutaneous tissue covering of the spinous process. Thus, the bone marrow within the spinous process in rats is exposed to the NIR irradiation.

Near-infrared irradiation dramatically increased subcutaneous adipocytes on the panniculus carnosus as well as CD34-positive cells surrounding the subcutaneous adipocytes. Histological analysis of the adipose tissue has revealed that CD34-positive cells are widely distributed among adipocytes and play a role in vascular stabilization.^7^ Adipogenesis is tightly associated with angiogenesis, and the expression of adipocytes is linked to the development of its vascularure.^8^ These reports suggest that NIR irradiation may stimulate CD34-positive cells to affect adipogenesis and angiogenesis on the panniculus carnosus.

Near-infrared irradiation resulted in an early increase in the number of bone marrow adipocytes^9^^-^10 and gradually increased the cortical bone mass. This was accompanied by an enrichment in CD34-positive stem cells especially at the inner surface of the bone cortex. CD34 is a marker for activated stem cells^11^ and is highly expressed on hematopoietic cells at the earliest stages of their development.^12^ The stem cell microenvironment (niche) is composed of mostly osteoblasts, which line the inner surface of bone and produce hematopoietic growth factors that regulate the formation and differentiation of hematopoietic stem cells.^13^,^14^ An increase in the number of osteoblasts results in a concurrent increase in the hematopoietic stem cell population and the cortical bone mass.^15^,^16^

Near-infrared irradiation dramatically decreased hematopoietic bone marrow cell counts by apoptosis. TUNEL identifies DNA fragmentation and can thus stain positive in cells undergoing either apoptosis or necrosis. In this study, we did not detect any evidence of necrosis. Therefore, positive TUNEL staining suggested that NIR irradiation induces a kind of apoptosis. NIR irradiation has previously been reported to induce strand breaks and cell death by apoptosis.^17^ Actively proliferating cells also show increased sensitivity to red and NIR radiation.^18^,^19^

To address the physiological relevance of our study, we can make the following calculations: The energy of the NIR irradiation we used was 40 J/cm^2^, each treatment consisted of 2 passes of NIR irradiation, and a total of 3 rounds of NIR treatment were performed on each rat. Thus, each rat was exposed to a total NIR energy of 40 × 2 × 3 J/cm^2^. The total incident solar energy at sea level is reported to be 0.0747 W/cm^2^.^20^ The energy distribution emitted by the NIR device used at wavelengths 1100 to 1800 nm is mathematically estimated to constitute 10.2% of total incident solar energy at sea level. Thus, incident NIR (1100–1800 nm) in solar energy will be 0.0076194 W/cm^2^. Thus, the energy emitted by these NIR treatments is approximately equivalent to sunbathing for 8 hours and 45 minutes in North America (40 [J/cm^2^] × 2 [pass] × 3 [round]/0.0076194 [W/cm^2^] = 31498.8188 [s]). Sunburn is believed to occur when skin has been burned by ultraviolet radiation, most often after prolonged exposure to the sun. However, non-thermal damages to the subcutaneous tissues can occur when skin is exposed to NIR radiation while sunbathing. Therefore, exposed skin should be protected with sunscreens that block not only ultraviolet but also NIR radiation.

Near-infrared irradiation damages superficial muscle^4^,^5^ as well as bone marrows cells. In contrast, it thickens the dermis and may enrich the stem cells that induce the number of adipose cells and increase cortical bone mass. These findings are relevant to facial skin that is constantly exposed to the sun as follows: solar NIR radiation may thicken the facial dermis, thereby increasing the number of superficial granular adipocytes and weakening the superficial facial muscles, which results in eyelid and facial skin ptosis. Although further studies are required to confirm our results, they may have major implications in facial aging.

## CONCLUSIONS

Near-infrared irradiation that simulated solar radiation non-thermally affected subcutaneous adipocytes and bones in rats. It induced putative, non-thermal damage of bone marrow, which was mediated by apoptosis. However, it increased subcutaneous and bone marrow adipocytes, CD34-positive hematopoietic stem cells in bone marrow, and cortical bone mass.

## Acknowledgments

We thank Ikuo Matsuyama for histological staining and the members of Cutera Inc for helpful comments.

**Disclosure**: None of the authors of this study have a conflict of interest.

## References

[1] Tanaka Y, Matsuo K, Yuzuriha S, Shinohara H (2009). Differential long-term stimulation of type I versus type III collagen after infrared irradiation. Dermatol Surg.

[2] Tanaka Y, Matsuo K, Yuzuriha S (2009). Long-term evaluation of collagen and elastin following infrared (1100 to 1800 nm) irradiation. J Drugs Dermatol.

[3] Tanaka Y, Matsuo K, Yuzuriha S (2010). Long-term histological comparison between near-infrared irradiated skin and scar tissues. J Clin Cosmet Invest Dermatol.

[4] Tanaka Y, Matsuo K, Yuzuriha S (2010). Long-lasting muscle thinning induced by infrared irradiation specialized with wavelength and contact cooling: a preliminary report. ePlasty.

[5] Tanaka Y, Matsuo K, Yuzuriha S (2011). Long-lasting relaxation of corrugator supercilii muscle contraction induced by near infrared irradiation. ePlasty.

[6] Tanaka Y, Matsuo K, Yuzuriha S, Yan H, Nakayama J (2010). Non-thermal cytocidal effect of infrared irradiation on cultured cancer cells using specialized device. Cancer Sci.

[7] Traktuev DO, Merfeld-Clauss S, Li J (2008). A population of multipotent CD34-positive adipose stromal cells share pericyte and mesenchymal surface markers, reside in a periendotherial location, and stabilize endothelial networks. Circ Res.

[8] Christiaens V, Lijnen HR (2010). Angiogenesis and development of adipose tissue. Review. Mol Cell Endocrinol.

[9] Srinivasan S, Pogue BW, Jiang S (2003). Interpreting hemoglobin and water concentration, oxygen saturation, and scattering measured *in vivo* by near-infrared breast tomography. Proc Natl Acad Sci USA.

[10] Van Veen RL, Sterenborg HJ, Pifferi A, Torricelli A, Chikoidze E, Cubeddu R (2005). Determination of visible near-IR absorption coefficients of mammalian fat using time- and spatially resolved diffuse reflectance and transmission spectroscopy. J Biomed Opt.

[11] Goodel MA (1999). CD34^+^ or CD34^−^: does it really matter?. Blood.

[12] Krause DS, Fackler MJ, Civin CI, May WS (1996). CD34: structure, biology, and clinical utility. Blood.

[13] Lemischka IR, Moore KA (2003). Interactive niches. Nature.

[14] Taichman RS, Emerson SG (1994). Human osteoblasts support hematopoiesis through the production of granulocyte colony-stimulating factor. J Exp Med.

[15] Zhang J, Niu C, Ye L (2003). Identification of the haematopoietic stem cell niche and control of the niche size. Nature.

[16] Calvi LM, Adams GB, Weibrecht KW (2003). Osteoblastic cells regulate the haematopoietic stem cell niche. Nature.

[17] Tirlapur UK, Knig K (2001). Femtosecond near-infrared laser pulse induced strand breaks in mammalian cells. Cell Mol Biol.

[18] Karu T, Pyatibrat L, Kalendo G (1994). Irradiation with He-Ne laser can influence the cytotoxic response of HeLa cells to ionizing radiation. Int J Radiat Biol.

[19] Tafur J, Mills PJ (2008). Low-intensity light therapy: exploring the role of redox mechanisms. Photomed Laser Surg.

[20] Gates DM (1966). Spectral distribution of solar radiation at the eart's surface. Science.

